# Development and Initial Psychometric Evaluation of the Adolescent Oral and Maxillofacial Health Knowledge–Attitude–Practice and Self-Efficacy Questionnaire (AOMH-KAPQ)

**DOI:** 10.3390/healthcare14162615

**Published:** 2026-08-19

**Authors:** Like Zhang, Xue Liu, Lei Cui, Xinyao Yang, Xinyue Hou, Lili Hou

**Affiliations:** 1 School of Nursing, Shanghai Jiao Tong University School of Medicine, Shanghai 200025, China; zhanglike@sjtu.edu.cn (L.Z.); xue-hang@sjtu.edu.cn (X.L.); srylayee@sjtu.edu.cn (L.C.); yangxinyao@sjtu.edu.cn (X.Y.); shjtdxhl2025hxy@sjtu.edu.cn (X.H.); 2 Department of Nursing, Shanghai Ninth People’s Hospital, Shanghai Jiao Tong University School of Medicine, Shanghai 200011, China

**Keywords:** adolescent, health knowledge, attitudes, practice, oral health, psychometrics, surveys and questionnaires

## Abstract

Background/Objectives: Existing oral-health questionnaires differ in scope from an instrument that combines objectively scored knowledge with separate attitude, practice, and self-efficacy domains across multiple adolescent oral and maxillofacial topics. This study developed the Adolescent Oral and Maxillofacial Health Knowledge–Attitude–Practice and Self-Efficacy Questionnaire (AOMH-KAPQ) and evaluated its initial psychometric properties. Methods: A 46-item pool derived from the KAP framework, literature, and clinical education content was refined through consultation with 18 experts and cognitive interviews with 10 adolescents. The questionnaire was administered to 1006 adolescents aged 12–18 years, and the sample was randomly divided for exploratory factor analysis (EFA) and confirmatory factor analysis (CFA; *n* = 503 each). Reliability, measurement invariance, and knowledge-item performance were also examined. Results: The final 38-item questionnaire comprised knowledge (15 items), attitude (9), practice (9), and self-efficacy (5). EFA identified one attitude and two provisional practice factors. In CFA, SRMR was ≤0.051 across dimensions; self-efficacy showed CFI = 0.991, TLI = 0.982, and RMSEA = 0.082, whereas robust RMSEA remained elevated for attitude (0.137) and practice (0.145). Cronbach’s α was 0.855–0.929, McDonald’s ω 0.867–0.930, and test–retest ICC 0.797–0.866. Conclusions: The AOMH-KAPQ showed good reliability in this sample, but structural evidence for the attitude and practice dimensions remains preliminary. At present, use should be limited to research and group-level educational needs assessment rather than individual clinical decisions.

## 1. Introduction

Oral health encompasses oral function, appearance, and psychosocial well-being [1,2]. Adolescence is a formative period in which health behaviors consolidate [3]. In China, dental caries affects the permanent dentition of 34.5% of 12-year-olds [4]. Adolescents may also experience malocclusion, dental trauma, temporomandibular disorders, and sleep-related breathing problems [5,6,7,8,9,10]. Recurrent aphthous stomatitis is another common oral mucosal condition [11]. Permanent-tooth loss may result from trauma or tooth agenesis, and implant rehabilitation in growing jaws requires cautious timing and follow-up [12]. These conditions differ in their warning signs and in the preventive or care-seeking responses they require; assessment should therefore extend beyond caries and routine oral hygiene alone.

The knowledge–attitude–practice (KAP) framework is commonly used to organize health-behavior assessment [13]. Oral-health studies have examined associations among knowledge, attitudes, practices, and oral outcomes [14,15], and KAP questionnaires have also been developed for other child and adolescent health behaviors [16]. However, weak mapping among knowledge, attitude, and practice components is a recognized limitation of KAP surveys [17]. The constructs and intended uses of existing standardized instruments differ from the measurement purpose of this study. The CPQ11–14 assesses oral-health-related quality of life, the PIDAQ assesses the psychosocial impact of dental esthetics, and the DC/TMD supports standardized assessment of temporomandibular disorders [18,19,20]. The OHBQAHBM assesses six Health Belief Model constructs related to adolescent oral-health behavior [21], whereas the KAB questionnaire reported by Selvaraj et al. assesses general oral-health knowledge, attitudes, and hygiene behaviors in adults [22]. In contrast, the AOMH-KAPQ was designed for adolescents and combines objectively scored knowledge with separate attitude, practice, and self-efficacy domains across six oral and maxillofacial topics. It was developed for this measurement purpose, not as a replacement for diagnostic, quality-of-life, oral-health-literacy, or single-topic instruments.

Health knowledge does not translate uniformly into behavior. Social-cognitive models identify self-efficacy as one factor shaping whether intentions are enacted [23]. Empirical studies have examined self-efficacy in general health promotion and in oral-health behavior among pre-university students [24,25]. An adolescent trial also examined self-efficacy as a mediator of intervention-related changes in toothbrushing and flossing [26]. These findings support measuring self-efficacy as a complementary construct, but they do not establish a fixed causal sequence among the AOMH-KAPQ dimensions.

The AOMH-KAPQ was designed as an adolescent-completed instrument for research and group-level needs assessment in school, community, and health-service settings. It covers six topics: dentofacial deformity, dental trauma, temporomandibular disorders, obesity-related sleep-apnea manifestations, persistent oral ulcers, and missing-tooth/restorative care. It organizes items within the KAP framework, scores knowledge objectively, and includes a separate self-efficacy module. This study aimed to develop the AOMH-KAPQ and evaluate its content validity, structural validity, reliability, measurement invariance, and knowledge-item performance in Chinese adolescents.

## 2. Materials and Methods

### 2.1. Design

**Figure 1:**
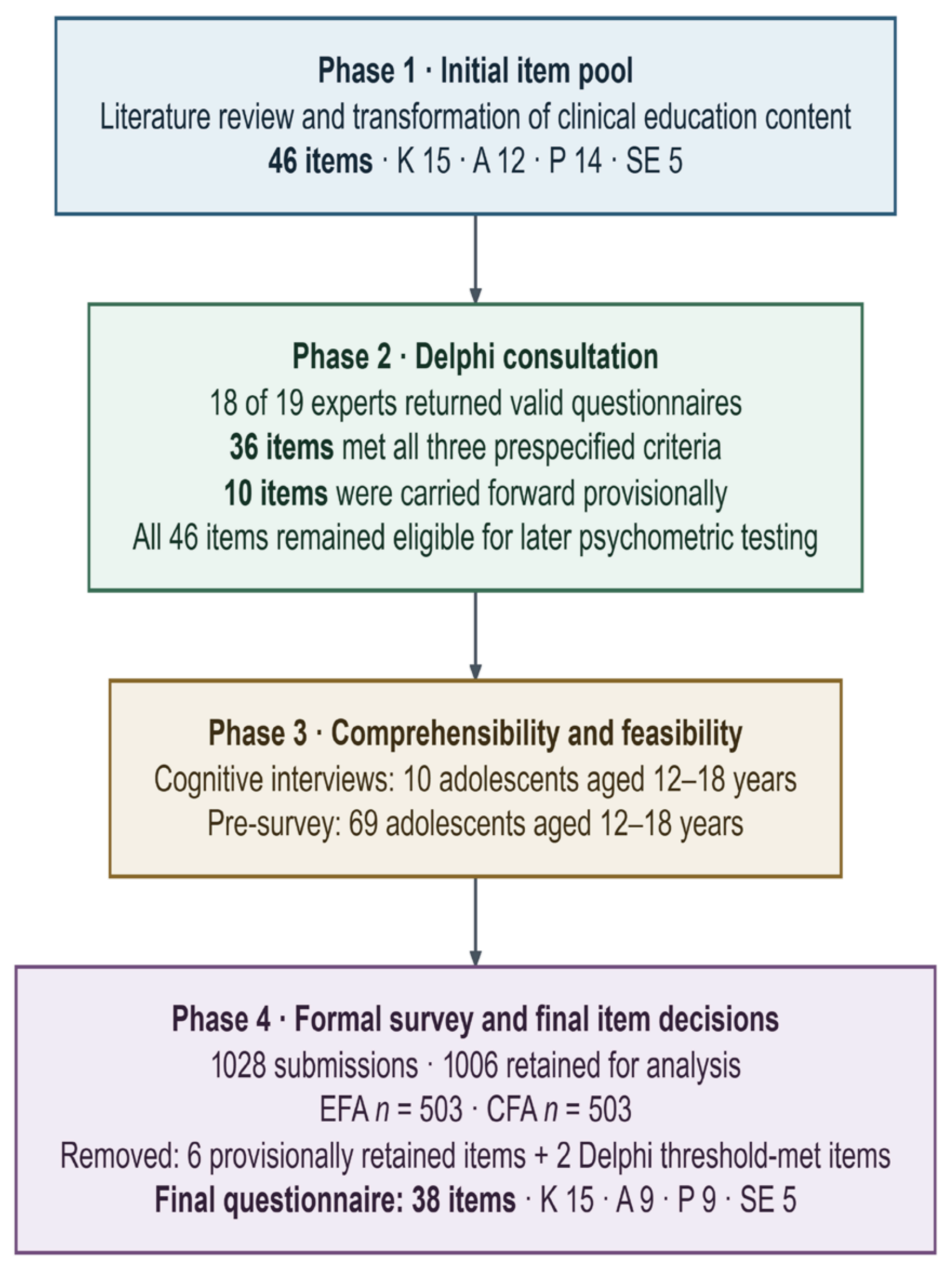
Item flow across development and initial psychometric evaluation of the AOMH-KAPQ. The 46-item pool entered formal testing after Delphi consultation, cognitive interviews, and a pre-survey; 8 items were removed during large-sample psychometric testing, leaving 38 final items. EFA = exploratory factor analysis; CFA = confirmatory factor analysis.

This cross-sectional methodological study followed the conventional sequence for questionnaire development [27,28]. It comprised four phases (Figure 1): (1) generation of an initial item pool; (2) item refinement through Delphi consultation and cognitive interviews; (3) a pre-survey; and (4) a formal multicenter survey and initial psychometric evaluation. The study was designed to obtain initial evidence for domain scores; it did not establish diagnostic cutoffs, a hierarchical overall score, or causal pathways among knowledge, attitude, practice, and self-efficacy.

### 2.2. Theoretical Framework and Dimensions

The KAP framework organized the questionnaire domains but was not treated as a causal model. Knowledge, attitude, and practice were operationalized as distinct but conceptually related domains [13]. Self-efficacy was included as a complementary social-cognitive construct because it captures perceived capability to perform the target behaviors [21,23]. The study did not prespecify a hierarchical total-score model or a mediation pathway among the four domains, and its cross-sectional design cannot establish causal direction. Accordingly, the AOMH-KAPQ yields separate domain scores; neither section order nor score associations should be interpreted as evidence that one dimension causes another. No existing standardized questionnaire served as the source measure; the AOMH-KAPQ was newly developed through item generation, expert review, cognitive interviewing, and psychometric testing in accordance with scale-development guidance [27,28].

Knowledge items assess recognition of correct and incorrect oral and maxillofacial health statements and are scored dichotomously (correct = 1; incorrect or not sure = 0). Attitude, practice, and self-efficacy items use five-point response scales developed for this study. The questionnaire yields four domain scores that should be reported and interpreted separately. The present evidence does not support an overall score. No low/moderate/high categories, diagnostic cutoff, or individual clinical-risk threshold was developed or validated. The item content spans six oral and maxillofacial topics, but the blueprint was not designed as a fully crossed topic-by-domain matrix, and topic-specific item patterns are descriptive rather than validated topic subscale scores.

### 2.3. Phase 1: Item Pool Generation

Literature search and item extraction. Chinese and English databases (CNKI, Wanfang, PubMed, and Web of Science) were searched from inception to August 2025. Knowledge, attitude, and behavior content from eligible literature was extracted and classified by KAP dimension.

Transformation of clinical health-education content. More than 400 health-education question-and-answer pairs compiled in relevant stomatological departments were reviewed. Two researchers independently screened, classified, and converted this content into candidate items, and disagreements were resolved by group discussion. Each item was written to capture a concrete warning sign, preventive action, or care-seeking cue rather than generic oral hygiene content. The final-item blueprint is shown in Table 1, and item sources and clinical cues are mapped in Supplementary Table S2.

**Initial item pool.** After literature extraction, clinical transformation, and several rounds of group discussion, an initial pool of 46 items was formed (knowledge 15, attitude 12, practice 14, self-efficacy 5).

### 2.4. Phase 2: Delphi Consultation and Cognitive Interviews

Experts were recruited by purposive sampling using study-specific eligibility criteria: (1) at least 10 years of work in orthodontics, oral and maxillofacial surgery, oral mucosal diseases, oral implantology, pediatric dentistry, nursing, or health communication; (2) an associate senior professional title or higher; and (3) provision of informed consent and the ability to complete the consultation.

Experts rated each item’s importance on a five-point scale and relevance to its assigned dimension on a four-point scale and provided suggestions on wording, response options, and dimension assignment. Prespecified retention required a mean importance score ≥ 4.0, a coefficient of variation (CV) < 0.25, and an item-level content validity index (I-CVI) ≥ 0.78 [29]. Decisions used unrounded values. Items that failed any criterion were not accepted as final at the Delphi stage. They were carried forward provisionally only when the research group judged that they covered nonredundant safety, prevention, or care-seeking content or that feedback identified a remediable wording or context problem. Provisional status meant entry into formal testing, not final acceptance, and all provisional items remained eligible for deletion.

The consultation record linked expert feedback to wording and context changes. Recorded examples included emphasizing prompt professional care while retaining the 30 min cue in K6; adding a lay description of snoring with breathing pauses in K10; specifying an ordinary nonviral ulcer in K13; adding growth and professional-evaluation context in K14; and limiting the mouthguard context in P5 to sports with physical contact or elevated dental-trauma risk.

**Indices.** The expert positive-response coefficient represented the effective return rate, and the authority coefficient (Cr) was calculated as the arithmetic mean of the judgment-basis coefficient (Ca) and familiarity coefficient (Cs). Kendall’s coefficient of concordance (Kendall’s W) measured agreement, and the CV indicated the dispersion of expert ratings.

Cognitive interviews. After item revision and before the formal survey, 10 adolescents aged 12–18 years (grades 6–12; five boys and five girls; stratified across municipality/provincial-capital, prefecture-level city, and county/township) underwent cognitive interviews. Online video interviews (8–12 min each) were audio-recorded and transcribed verbatim. Verbal probing [30] was used to examine comprehension of technical terms, interpretation of scenario-based items, discrimination among Likert response options, and the reasoning used to answer reverse-scored items. Item wording and response options were revised accordingly.

### 2.5. Phase 3: Pre-Survey

In October 2025, 69 adolescents aged 12–18 years completed a pre-survey to assess readability, item comprehension, ease of completion, and completion time; the data were not used for statistical item screening or reliability estimation. The median completion time was approximately 4 min, all required items were completed, and the questionnaire length was considered acceptable for adolescents.

### 2.6. Phase 4: Formal Survey and Psychometric Testing

Participants and sampling. Between January and March 2026, a multicenter cross-sectional survey used convenience sampling through a national oral-health alliance network led by a tertiary stomatological hospital in Shanghai. Inclusion criteria were (1) current school enrollment; (2) age 12–18 years; (3) sufficient Chinese reading comprehension; and (4) parent/legal-guardian electronic consent together with adolescent assent. The exclusion criterion was a serious psychiatric or cognitive condition preventing questionnaire completion. The survey platform recorded 1028 complete submissions. Available project records indicate that response duration, patterned responding, and possible duplicate submissions were considered jointly during data-quality review. This review excluded 22 submissions, leaving 1006 questionnaires for analysis. Because the contemporaneous case-level adjudication log was incomplete, no individual screening threshold is reported as a mechanically applied exclusion rule. The study protocol used a planning range of 5–10 respondents per item; for the 46-item pool, this corresponded to 230–460 participants, with allowance for invalid responses. Scale-development guidance recommends approximately 10 respondents per item and/or 200–300 observations [28]. The final sample of 1006 exceeded these benchmarks and was randomly divided into EFA and CFA subsets of 503 participants each.

Data collection and quality control. Electronic questionnaires were distributed via the Wenjuanxing platform through WeChat QR codes under a standardized operating procedure at each center. The electronic form required responses to all psychometric items, and the retained analysis dataset contained no missing item-level values.

### 2.7. Statistical Analysis

Analyses were performed using IBM SPSS Statistics for Windows, version 26.0 (IBM Corp., Armonk, NY, USA), and R, version 4.5.3 (R Foundation for Statistical Computing, Vienna, Austria), with the lavaan (version 0.6-21), psych (version 2.6.5), semTools (version 0.5-8), and mirt (version 1.46.1) packages. The 1006 cases were randomly divided into an EFA half (*n* = 503) and a CFA half (*n* = 503); the halves did not differ in self-reported gender, age, or school stage (all *p* > 0.05). The significance level was α = 0.05.

Use of generative artificial intelligence during revision. OpenAI Codex (GPT-5; OpenAI, accessed July 2026) was used to assist with drafting reproducible R code for reviewer-requested CFA sensitivity analyses and with document quality control. Generative AI was not used to generate or alter study data. Reported numerical results were checked against the saved model outputs; analytical decisions and interpretation remain the authors’ responsibility.

*Item analysis.* Knowledge items were evaluated using item difficulty and point-biserial correlations. Practice items referred to the previous six months; when respondents had not encountered a conditional scenario, they were instructed to answer how they would respond. Consequently, practice-domain scores combine reported behavior with intended conditional responses. Attitude, practice, and self-efficacy items were evaluated using the critical-ratio method, corrected item–total correlations (CITCs), and changes in α after item deletion.

Validity. Structural validity of the attitude and practice dimensions was examined by EFA and CFA, whereas self-efficacy was examined by CFA; the dichotomously scored knowledge dimension was evaluated with KR-20 and a two-parameter IRT model rather than factor-analyzed. EFA used principal axis factoring; the number of factors was informed by eigenvalues, the scree plot, parallel analysis [31], and interpretability, with oblique rotation for multifactor solutions. Ordered-categorical CFA used the robust weighted least squares mean- and variance-adjusted (WLSMV) estimator. Model evaluation jointly considered scaled χ^2^ and degrees of freedom, robust CFI, TLI, RMSEA, and SRMR, standardized loadings, convergence, and substantive interpretability. The protocol prespecified >0.90 for CFI/TLI and <0.08 for RMSEA/SRMR; because cutoff performance depends on the estimator and model conditions, no index was used as a stand-alone decision rule [32,33]. For attitude, A2 was removed after a modification index (MI = 12.061) indicated local dependence and high content overlap with A3 in the CFA half. Because the resulting nine-item attitude model was refined and evaluated in the same CFA half, it is treated as same-sample evidence requiring independent confirmation. For practice, the EFA-derived two-factor model was first compared with a single-factor model. A residual covariance between P6 and P8 was then added because both items involve informing a parent and seeking professional care. Nested WLSMV models were compared with scaled difference tests. Because the P6–P8 refinement was evaluated in the same CFA half, the retained practice model was treated as provisional and requiring independent replication. Convergent and discriminant validity were examined with AVE, CR, the Fornell–Larcker comparison [34], and HTMT [35]. Sensitivity analyses used the original CFA split and final item coding. The WLSMV models were re-estimated with delta and theta parameterizations, with ULSMV, and with robust maximum likelihood (MLR; treating five-category items as continuous). For attitude, content-based residual covariances were tested for A7–A8, A4–A6, and both pairs, together with a substantively specified bifactor model. These were sensitivity tests rather than data-driven searches for improved fit.

Reliability. Cronbach’s α (>0.70 considered acceptable [27]) and McDonald’s ω [36] were used to assess internal consistency, the Spearman–Brown coefficient to assess split-half reliability, and ICC(2,1) to assess temporal stability.

Additional analyses. Ceiling and floor effects were examined, and measurement invariance across self-reported gender and age groups was evaluated using multi-group CFA, with ΔCFI ≤ 0.010 and ΔRMSEA ≤ 0.015 as criteria [37]. Model identification for ordered-categorical items followed Wu and Estabrook [38]. A two-parameter logistic IRT model (2PL) was also fitted as an exploratory item-level analysis of the knowledge dimension [39].

### 2.8. Ethical Considerations

The study was approved by the Ethics Committee of Shanghai Ninth People’s Hospital, Shanghai Jiao Tong University School of Medicine (No. SH9H-2026-TK697-1). Electronic informed consent was obtained from a parent or legal guardian, and assent was obtained from the participating adolescents before data collection.

## 3. Results

### 3.1. Participant Characteristics

A total of 1006 valid responses were analyzed (EFA half *n* = 503; CFA half *n* = 503). Participants were 12–18 years old. Self-reported gender was recorded using the questionnaire’s original binary response options (male/female); characteristics are shown in Table 2.

### 3.2. Delphi Consultation

Nineteen experts were invited, and 18 returned valid questionnaires (positive-response coefficient 94.7%). The authority coefficient was 0.90 (Ca = 0.88, Cs = 0.92). Across the 46 items, I-CVI ranged from 0.61 to 1.00, S-CVI/Ave was 0.92, the CV of importance ratings ranged from 0.08 to 0.30 (mean 0.17), and tie-corrected Kendall’s W was 0.202 (χ^2^ = 163.64, df = 45, *p* < 0.001). Using unrounded values, 36 items met all three prespecified criteria. Ten items (K13, K14, A5, A11, P4, P5, P7, P9, P10, and P12) failed at least one criterion and were carried forward provisionally rather than accepted as final. During formal psychometric testing, six of these provisional items (A5, A11, P4, P7, P9, and P12) were removed, whereas K13, K14, P5, and P10 remained. The retained provisional knowledge items showed acceptable 2PL performance: K13 (a = 1.08, b = 0.11) and K14 (a = 1.25, b = 0.27). The retained provisional practice items also performed acceptably in the large-sample analyses: P5 (loading = 0.621, CITC = 0.400) and P10 (loading = 0.642, CITC = 0.733). Their final retention therefore reflected both nonredundant content coverage and acceptable later-stage empirical performance, not content importance alone. Two additional items that met the Delphi criteria (A2 and P1) were removed after large-sample testing. Table 3 summarizes dimension-level results, and Supplementary Table S1 gives each initial item’s mean importance, CV, I-CVI, Delphi-stage decision, and final status.

### 3.3. Item Analysis

The 15 binary knowledge items had a KR-20 of 0.86 and corrected point-biserial correlations of 0.27–0.68. K12 (0.27) and K6 (0.29) were relatively low, but deleting either changed KR-20 only marginally; both were retained because they represented safety-relevant, nonredundant content. In attitude, reverse-scored A5 and A11 were deleted for weak item–total consistency, and A2 was subsequently deleted during CFA-sample refinement after a modification index (MI = 12.061) indicated local dependence and high content overlap with A3, leaving 9 items. In practice, reverse-scored P4, P7, and P12 were deleted, and P1 (loading < 0.40) and P9 (salient cross-loading) were further removed, leaving 9 items (Table 4). After these eight deletions, the final 38-item questionnaire retained all six oral and maxillofacial topics and all four score domains at the instrument level (Table 1), although representation was unequal across topic-by-domain cells.

### 3.4. Exploratory Factor Analysis

In the EFA half (*n* = 503), the 10 attitude items remaining after removal of A5 and A11 yielded a KMO of 0.94, and Bartlett’s test was significant (*p* < 0.001). The first and second eigenvalues were 7.25 and 0.74, respectively. The scree plot flattened after the first factor, and a single factor was extracted, accounting for 58.8% of the variance with loadings of 0.65–0.88. The nine practice items remaining after removal of P4, P7, P12, P1, and P9 yielded a KMO of 0.88, and Bartlett’s test was significant (*p* < 0.001). The first three eigenvalues were 5.13, 1.33, and 0.68. The scree plot, parallel analysis, and item interpretability supported a two-factor solution, which accounted for 54.7% of the variance after oblique rotation (inter-factor r = 0.50). Factor 1 (condition-responsive self-care and care-seeking) comprised P6, P8, P10, P11, P13, and P14, and Factor 2 (routine preventive practices) comprised P2, P3, and P5. The loadings are shown in Table 5; these labels describe the observed item groupings and do not establish separately validated practice subscales.

### 3.5. Confirmatory Factor Analysis and Competing Models

In the CFA half (*n* = 503), attitude was modeled as a nine-item single-factor model, practice as a nine-item two-factor model with a residual covariance between P6 and P8, and self-efficacy as a five-item single-factor model (Table 6). For attitude, CFI (0.946), TLI (0.928), and SRMR (0.030) met the prespecified thresholds, but robust RMSEA was 0.137. For practice, CFI (0.913) and SRMR (0.051) met the thresholds, whereas TLI (0.875) and robust RMSEA (0.145) did not. For self-efficacy, CFI, TLI, and SRMR met the prespecified thresholds, while RMSEA (0.082) was just above 0.08. The evidence was therefore evaluated as mixed rather than classified by any single fit index.

**Figure 2:**
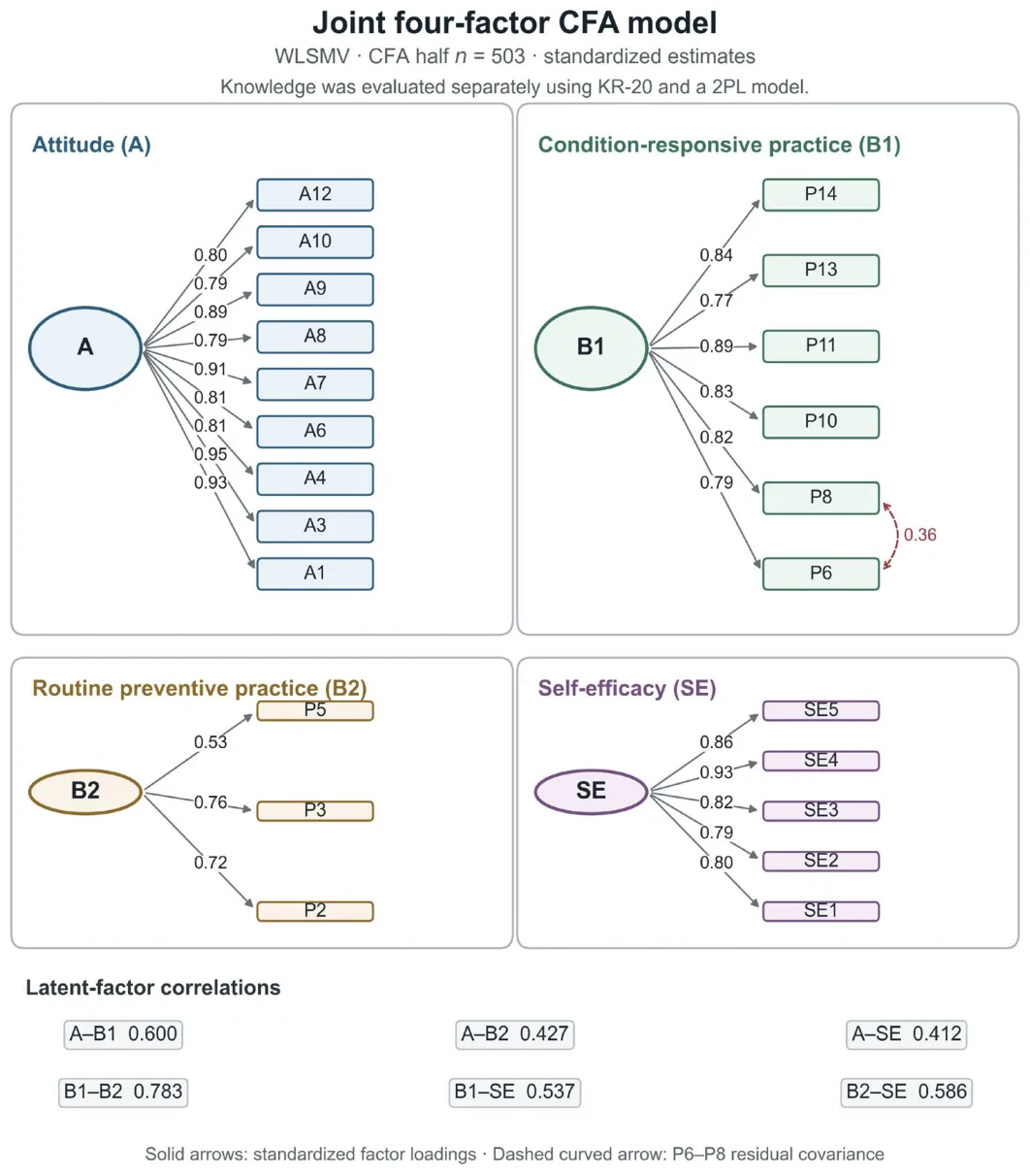
Confirmatory factor analysis of the AOMH-KAPQ: joint four-factor measurement model (WLSMV, CFA half *n* = 503). Ovals are latent factors (A = attitude; B1 = practice, condition-responsive self-care and care-seeking; B2 = practice, routine preventive practices; SE = self-efficacy) and rectangles are items. Single-headed arrows show standardized factor loadings; the dashed curved arrow linking P6 and P8 is a residual covariance. Latent-factor correlations are displayed below the path diagram and reported in Supplementary Table S6. The dichotomously scored knowledge domain was evaluated separately and is not part of this latent model. Robust fit: CFI = 0.905, TLI = 0.893, RMSEA = 0.094, SRMR = 0.047.

Table 6 reports the comparisons among the practice models. The model used in subsequent analyses included the P6–P8 residual covariance, whereas the practice bifactor model did not converge to an admissible solution. Because the residual covariance was evaluated in the same CFA half, this model refinement remains provisional. The joint four-factor WLSMV model yielded CFI = 0.905, TLI = 0.893, RMSEA = 0.094, and SRMR = 0.047. CFI and SRMR met the prespecified thresholds, whereas TLI and RMSEA did not; the joint model was therefore treated as supplementary rather than confirmatory evidence (Figure 2).

Re-estimating the WLSMV models using theta rather than delta parameterization yielded identical fit indices. ULSMV did not improve robust RMSEA for attitude, practice, or the joint model (0.139, 0.145, and 0.096, respectively). MLR reduced RMSEA to 0.110, 0.096, and 0.060; only the joint MLR model fell below 0.08, whereas the separate attitude and practice models did not. Content-based attitude residual-covariance models yielded robust RMSEA values of 0.141–0.144, and the attitude bifactor model did not converge. No defensible alternative resolved the separate-dimension misfit; complete results are reported in Supplementary Table S5.

### 3.6. Convergent and Discriminant Validity

Table 7 summarizes the principal measurement findings and their interpretation for nontechnical readers. Full convergent/discriminant and measurement-invariance results are reported in Supplementary Tables S6 and S7. Per-item knowledge IRT estimates and standard errors are reported in Supplementary Table S3, with an interpretive summary in Table S8.

In the joint measurement model (Supplementary Table S6), AVE was 0.732 for attitude, 0.679 for practice B1 (condition-responsive self-care and care-seeking), 0.457 for practice B2 (routine preventive practices), and 0.709 for self-efficacy; corresponding CRs were 0.961, 0.927, 0.712, and 0.924. Evidence for separating the practice factors was mixed. Although HTMT was 0.800, the B1–B2 correlation was 0.783 and exceeded √AVE for B2 (0.676), and B2 contained only three items. The two factors should therefore be interpreted as provisional descriptive components within the broader practice domain rather than as independently validated subscales.

### 3.7. Reliability

In the full sample (*n* = 1006), Cronbach’s α ranged from 0.855 to 0.929 across the knowledge, attitude, practice, and self-efficacy dimensions; McDonald’s ω ranged from 0.867 to 0.930; and split-half reliability ranged from 0.870 to 0.940 (Table 8). Among 58 adolescents retested after approximately 14 days, dimension-level ICC(2,1) values ranged from 0.797 to 0.866. These coefficients indicate good internal consistency and temporal stability for each domain. They do not support an overall score across the four differently scored domains.

### 3.8. Ceiling and Floor Effects

For knowledge, floor and ceiling effects were 4.2% and 4.4%, respectively; the corresponding values for practice were 0.7% and 8.4%. All four values were below the 15% criterion. Ceiling effects for attitude (18.8%) and self-efficacy (19.6%) exceeded the criterion. Although only 4.4% obtained the maximum knowledge score, most knowledge-item difficulty parameters were below zero, indicating a relatively easy item set with reduced precision among adolescents at the high-knowledge end.

### 3.9. Measurement Invariance

In multi-group CFA, changes in fit across the configural, metric, and scalar models remained below the recommended thresholds for self-reported gender (male/female) and age group (ΔCFI ≤ 0.010; ΔRMSEA ≤ 0.015; Supplementary Table S7). These results support comparisons by self-reported gender and age group within this sample. They do not establish measurement equivalence in other geographic or cultural populations or support individual diagnosis.

### 3.10. Item Response Theory for the Knowledge Dimension

The 2PL model of the knowledge dimension showed acceptable fit (M2 test: RMSEA = 0.08, CFI = 0.95, TLI = 0.94) and an empirical reliability of 0.82. Item discriminations (a) ranged from 0.55 to 3.58 (most > 1) and difficulties (b) from −1.82 to 0.66 (most < 0, indicating relatively easy items; Supplementary Tables S3 and S8). K6 (a = 0.58) and K12 (a = 0.55) had the lowest discrimination, consistent with their low item–total correlations under classical test theory. Because the knowledge dimension spans several topics, these 2PL results are interpreted as exploratory item-level information rather than evidence of strict unidimensionality.

## 4. Discussion

The development sequence combined literature review, transformation of clinical health-education content, expert consultation, cognitive interviewing, pre-survey testing, and large-sample psychometric evaluation [28]. Item retention followed sequential evidence rather than an automatic statistical rule. Delphi ratings classified items by consensus status, cognitive interviews addressed comprehension, and item/factor analyses informed final retention. Conceptual relevance did not supersede subsequent psychometric evidence: six weak-consensus items and two Delphi-passing items were ultimately deleted. Conversely, K6 and K12 were retained despite limited discrimination because they represent time-critical dental-trauma and persistent-ulcer content. This choice preserved safety-relevant coverage but may make these retention decisions sample dependent, reinforcing the need for independent external validation.

Internal consistency and temporal stability were good across domains. The two practice factors may reflect different behavioral contexts: B1 groups condition-responsive self-care and care-seeking items, whereas B2 groups routine preventive practices. However, B1 and B2 were strongly related (r = 0.783; HTMT = 0.800), and B2 contained only three items with an AVE of 0.457. Moreover, √AVE for B2 (0.676) was below the B1–B2 correlation. The factors should therefore be treated as descriptive components within the broader practice domain, not as independently validated subscales or as a basis for individual decisions. Interpreting B1 and B2 as separate scores would require independent replication and additional condition-specific preventive items before inferential use.

The CFA evidence was mixed. CFI and SRMR met the prespecified thresholds for attitude and practice, and TLI met the threshold for attitude; however, robust RMSEA remained elevated for both dimensions. Alternative parameterizations and estimators, content-based residual-covariance models, and substantively specified bifactor models did not resolve the separate-dimension misfit. The nine-item attitude model was also refined in the CFA half through deletion of A2, and the P6–P8 residual covariance was evaluated in that same half; both decisions therefore require independent replication. The improved joint-model fit under MLR does not override the ordered-categorical WLSMV findings. The attitude and practice structures are therefore provisional pending confirmation in an independent sample; self-efficacy showed stronger structural evidence.

Recent KAP questionnaire development has likewise used IRT for objectively scored knowledge items and factor analysis for Likert-type attitude and practice items [40]. A dental psychometric study also reported subscale-specific differences in measurement performance rather than treating validity as a single instrument-wide property [41]. These examples support domain-specific reporting of the AOMH-KAPQ evidence; they do not remove the present attitude and practice limitations.

For knowledge items K6 and K12, classical test theory and IRT both indicated limited discrimination. They were retained because they measure the time-critical window for dental-trauma care and the need for assessment when an oral ulcer persists. The IRT results also showed that the knowledge items were generally easy, limiting discrimination among high-knowledge respondents; future revisions should test more difficult items. Because the knowledge dimension spans multiple topics, its 2PL results remain exploratory item-level information rather than evidence of strict unidimensionality.

Within the tested Likert-domain joint model, changes in CFI and RMSEA met the prespecified metric and scalar invariance criteria across self-reported gender and age groups [37]. The ordered-categorical invariance models followed the identification approach described by Wu and Estabrook [38]. These relative invariance results are conditional on the provisional latent structure and mixed absolute fit of the joint model; they do not cover the knowledge domain. Independent geographic and cultural replication remains necessary.

At the group level, dimension scores and topic-specific item patterns may indicate whether education should prioritize, for example, avulsed-tooth first aid, persistent-ulcer care-seeking, or sports mouthguard use. The questionnaire is completed by adolescents. Researchers and health professionals, including nurses, may use the resulting group-level patterns to plan education. Repeated administration before and after a program may be used to describe group-level changes in knowledge, attitudes, practice-domain responses, and self-efficacy. Responsiveness has not yet been established; however, such changes should be interpreted as exploratory and not as evidence of individual clinical improvement.

### Limitations

Several sampling and data-collection limitations should be considered. Participants were recruited by convenience through a national oral-health alliance network and completed an online questionnaire. The sites did not constitute a probability-based national sample; selection bias, coverage bias, and limited geographic representativeness therefore preclude generalization to all Chinese adolescents. Responses in the attitude, practice, and self-efficacy domains were self-reported and may be affected by social-desirability bias. The six-month practice reference period may also introduce recall bias. Although the reduction from 1028 submissions to 1006 analyzed questionnaires is documented, the retained project files did not contain a complete contemporaneous case-level adjudication log for the 22 excluded submissions, which limits the auditability of data-quality screening. Several practice items were conditional on events that some adolescents had not encountered; these respondents were instructed to answer how they would respond. The practice score therefore combines recalled behavior with hypothetical conditional responses and should not be interpreted as the frequency of observed behavior alone.

The psychometric evidence also has important limits. The EFA and CFA subsets were random halves of the same survey rather than independent cohorts, and robust RMSEA remained elevated for attitude and practice despite the sensitivity analyses. A2 was deleted and the final nine-item attitude model was evaluated within the same CFA half; likewise, the P6–P8 residual covariance was added and evaluated within that half. Both refinements may therefore be sample dependent and require independent replication. Most knowledge items were relatively easy, which limits high-end precision even though the maximum-score percentage was only 4.4%. Ceiling effects for attitude (18.8%) and self-efficacy (19.6%) may limit discrimination and responsiveness at higher levels. The retest sample included 58 adolescents, and practice B2 contained only three items with an AVE of 0.457 and incomplete discriminant separation from B1. The study did not evaluate criterion-related validity against clinical examination or observed behavior, diagnostic accuracy, or responsiveness. Current use should therefore remain limited to research and group-level educational needs assessment pending independent validation.

## 5. Conclusions

In this sample, the AOMH-KAPQ yielded initial psychometric evidence for four separately interpreted domain scores while retaining coverage of six oral and maxillofacial topics. Internal consistency and short-term stability were good, whereas structural evidence was mixed, particularly for attitude and practice; those structures remain provisional. The adolescent-completed questionnaire may support research and group-level educational needs assessment in populations similar to the study sample, but it should not be used for individual clinical decisions, diagnosis, or risk classification. Independent validation across geographic, demographic, and cultural contexts, together with criterion-related, clinical, and responsiveness evidence, is needed before broader application.

## Figures and Tables

**Table 1:** Questionnaire blueprint: Distribution of items across the six oral and maxillofacial topics and the four dimensions.

Oral–Maxillofacial Topic	Knowledge (K)	Attitude (A)	Practice (P)	Self-Efficacy (SE)
Dentofacial deformity/malocclusion	K1, K2, K3	A3	—	—
Dental trauma	K4, K5, K6	A4, A6	P6, P5	SE3
Temporomandibular disorders (TMD)	K7, K8	A7, A8	P8, P3	—
Obesity-related OSA manifestations	K9, K10	A9	P10	—
Oral ulcers	K11, K12, K13	A10	P11, P13	—
Missing teeth/restorative timing	K14, K15	A12	P14 †	SE5
General/cross-cutting	—	A1	P2	SE1, SE2, SE4

Note: The final 38 items retain all six oral and maxillofacial topics and all four score domains at the instrument level; the blueprint is not a fully crossed topic-by-domain matrix, and several cells are empty or represented by few items. Full wording is given in Table S4. † P14 concerns post-implant maintenance and is context dependent; broader restorative/appliance wording should be tested in future versions.

**Table 2:** Characteristics of the participants (*N* = 1006).

Variable	Category	*n*	%
Self-reported gender	Male	426	42.3
	Female	580	57.7
Age (years)	12–15	502	49.9
	16–18	504	50.1
School stage	Primary	67	6.7
	Junior high	429	42.6
	Senior high	510	50.7

**Table 3:** Content validity index and coefficient of variation by dimension (18 experts, 46 items).

Dimension	Items	Mean I-CVI	I-CVI Range	Mean CV	CV Range
Knowledge (K)	15	0.933	0.722–1.000	0.163	0.114–0.257
Attitude (A)	12	0.949	0.611–1.000	0.174	0.079–0.302
Practice (P)	14	0.849	0.611–1.000	0.182	0.104–0.293
Self-efficacy (SE)	5	1.000	1.000–1.000	0.133	0.104–0.155

Note: I-CVI = item-level content validity index; CV = coefficient of variation.

**Table 4:** Item screening and decisions.

Dimension	Item	Statistical Performance	Basis	Decision
Attitude	A5 (reverse)	CITC = 0.158; α after removal = 0.901	Poor consistency	Deleted
Attitude	A11 (reverse)	CITC = 0.156; α after removal = 0.902	Poor consistency	Deleted
Attitude	A2	High overlap with A3 (MI = 12.06)	Content redundancy	Deleted
Practice	P4 (reverse)	CITC = −0.059; α after removal = 0.805	Inconsistent direction	Deleted
Practice	P7 (reverse)	CITC = −0.203; α after removal = 0.817	Inconsistent direction	Deleted
Practice	P12 (reverse)	CITC = −0.190; α after removal = 0.818	Inconsistent direction	Deleted
Practice	P1	EFA loading 0.346/0.239	Low loading	Deleted
Practice	P9	EFA loading 0.382/0.400	Cross-loading	Deleted

Note: CITC = corrected item–total correlation; MI = modification index.

**Table 5:** Exploratory factor analysis of the attitude and practice dimensions (*n* = 503) ^a^.

**Panel A—Attitude (Single Factor)**				
**Item**	**Abbreviated Content ^d^**		**Factor 1**	**Communality**
A3	Malocclusion should be managed at the right time on professional advice		**0.882**	0.778
A1	Knowing about oral and maxillofacial diseases is necessary		**0.863**	0.745
A2 ^c^	Crossbite/malocclusion should be examined and assessed early		**0.846**	0.716
A7	Recurrent or painful jaw clicking warrants attention		**0.832**	0.692
A12	A missing permanent tooth should be evaluated for restoration early		**0.731**	0.534
A9	Marked snoring or daytime sleepiness may signal a health problem		**0.724**	0.524
A4	A knocked-out tooth may be saved if handled properly		**0.719**	0.517
A6	A mouthguard during sport reduces the risk of dental trauma		**0.708**	0.501
A8	Stress can aggravate bruxism and cause jaw soreness		**0.668**	0.446
A10	Spicy/hard foods should be avoided during a mouth ulcer		**0.653**	0.426
*Initial eigenvalue*			7.25	
*Variance explained (%)*			58.8	
**Panel B—Practice (two factors, oblique rotation; inter-factor r = 0.502 ^b^** **)**				
**Item**	**Abbreviated content ^d^**	**Factor 1**	**Factor 2**	**Communality**
P14	Maintain cleaning after implant restoration	**0.859**	—	0.668
P11	Ulcer at the same site >2 weeks: tell parents, seek care	**0.850**	—	0.726
P8	Jaw clicking with pain >2 weeks: request care	**0.787**	—	0.625
P6	After a tooth is knocked out, tell parents and seek care	**0.698**	—	0.572
P13	Avoid spicy/hard foods when an ulcer occurs	**0.691**	—	0.436
P10	Severe snoring with sleepiness: tell parents, seek care	**0.642**	—	0.580
P2	Use floss or an interdental brush to clean between teeth	—	**0.771**	0.591
P5	Wear a mouthguard during contact sport	—	**0.621**	0.376
P3	Avoid long-term unilateral chewing	—	**0.471**	0.351
*Initial eigenvalue*		5.13	1.33	
*Cumulative variance (%)*			54.7	

Note: ^a^ AOMH-KAPQ = Adolescent Oral and Maxillofacial Health Knowledge–Attitude–Practice and Self-Efficacy Questionnaire. ^b^ Principal axis factoring; Panel B used direct oblimin rotation. Salient loadings are shown in boldface; loadings < 0.30 are shown as “—”. Factor 1 (practice) = condition-responsive self-care and care-seeking; Factor 2 = routine preventive practices. The labels describe item content and do not establish separately validated subscales. ^c^ A2 was removed at the CFA stage for high overlap with A3. ^d^ Item content is abbreviated for the table; the full English wording of all 38 items is given in Table S4.

**Table 6:** Confirmatory factor analysis model fit by dimension (*n* = 503).

Model	Scaled χ^2^	df	CFI	TLI	RMSEA	SRMR	Standardized Loadings
Reference value	—	—	>0.90	>0.90	<0.08	<0.08	—
Model A (attitude, 9 items, single-factor)	341.07	27	0.946	0.928	0.137	0.030	0.790–0.954
Practice, 9 items, single-factor (alternative)	403.22	27	0.873	0.831	0.168	0.064	—
Practice, 9 items, two factors without residual covariance	284.17	26	0.897	0.857	0.154	0.053	—
Practice, two factors + P6–P8 residual (retained)	232.36	25	0.913	0.875	0.145	0.051	0.589–0.907
Practice, 9 items, bifactor (alternative)	—	—	—	—	—	—	Did not converge
Model SE (self-efficacy, 5 items, single-factor)	24.20	5	0.991	0.982	0.082	0.018	0.797–0.908

Note: Scaled χ^2^ values are the WLSMV model-test statistics reported for transparency; model evaluation was based on robust CFI, TLI, and RMSEA together with SRMR, standardized loadings, convergence, substantive interpretability, and sensitivity analyses. The practice two-factor model without a residual covariance fit better than the single-factor model (scaled Δχ^2^ = 50.79, Δdf = 1, *p* < 0.001). Adding the P6–P8 residual covariance further improved fit (scaled Δχ^2^ = 60.28, Δdf = 1, *p* < 0.001), but this same-sample refinement requires independent replication. The practice bifactor model did not converge to an admissible solution. CFI = comparative fit index; RMSEA = root mean square error of approximation; SRMR = standardized root mean square residual; TLI = Tucker–Lewis index; WLSMV = weighted least squares mean- and variance-adjusted estimator.

**Table 7:** Reader-oriented summary of measurement evidence and interpretation.

Measurement Question	Key Result	Reader-Facing Meaning	Required Limitation
Is the structure clear?	Self-efficacy showed stronger structural evidence; attitude and practice evidence was mixed.	The four domain scores should be interpreted separately.	The attitude and practice structures require confirmation in an independent sample.
Are scores reliable?	α/ω = 0.855–0.930; ICC(2,1) = 0.797–0.866.	Dimension scores showed good consistency and short-term stability.	Reliability does not establish diagnostic accuracy or responsiveness.
Are group comparisons interpretable?	For the tested Likert-domain joint model, relative metric and scalar invariance criteria were met across self-reported gender and age groups.	Conditional comparisons of the tested latent structure are supported in this sample.	The knowledge domain was not included; the joint model had mixed absolute fit, and geographic/cultural invariance is unestablished.
Do knowledge items distinguish respondents?	Most discrimination parameters were moderate or high; most difficulty parameters were below zero.	The items distinguish general knowledge levels in the study sample.	The relatively easy item set provides less information at the high-knowledge end.

Note: Per-item 2PL estimates are reported in Supplementary Table S3; full coefficients and model-specific results for the Likert domains are reported in Supplementary Tables S5–S8. α = Cronbach’s alpha; ω = McDonald’s omega; ICC = intraclass correlation coefficient.

**Table 8:** Reliability by dimension: Are scores internally consistent and stable over time?

Dimension	Items	Cronbach’s α	McDonald’s ω	Split-Half	Test–Retest ICC
Knowledge (K)	15	0.855	0.867	0.903	0.798
Attitude (A)	9	0.929	0.930	0.940	0.797
Practice (P)	9	0.873	0.896	0.917	0.866
Self-efficacy (SE)	5	0.884	0.885	0.870	0.822

Note: Knowledge is dichotomously scored; its reported α is equivalent to KR-20. The test–retest analysis used ICC(2,1) over approximately 14 days in 58 adolescents. These coefficients support group-level score consistency and temporal stability but do not establish diagnostic accuracy or justify individual clinical decisions.

## Data Availability

The data analyzed in this study are not publicly available because the participants were minors and the ethics approval and informed consent did not provide for open data sharing. De-identified data may be made available by the corresponding author on reasonable request, subject to approval by the Ethics Committee of Shanghai Ninth People’s Hospital and a data-use agreement that safeguards participant privacy. Requests to access the datasets should be directed to Lili Hou (houlili@sjtu.edu.cn).

## Supplementary Materials

The following supporting information can be downloaded at: https://www.mdpi.com/article/10.3390/healthcare14162615/s1, Table S1: Item-Level Delphi Results, Delphi-Stage Decisions, and Final Status for All 46 Initial Items; Table S2: Item-Source and Specificity Mapping for the Retained 38 Items; Table S3: Two-Parameter (2PL) IRT Item Parameters for the 15 Knowledge Items (Full Sample, *N* = 1006); Table S4: The AOMH-KAPQ (final 38 items); Table S5: CFA Estimator, Parameterization, and Alternative-Model Sensitivity Analyses (CFA Half, N = 503); Table S6: Convergent and Discriminant Validity in the Joint Measurement Model (CFA Half, N = 503); Table S7: Measurement Invariance Across Self-Reported Gender and Age Groups (CFA Half, N = 503); Table S8: Interpretive Summary of Knowledge-Domain 2PL Results (Full Sample, N = 1006; Per-Item Estimates and Standard Errors in Table S3).
