# Supplementary material for: Development and Initial Psychometric Evaluation of the Adolescent Oral and Maxillofacial Health Knowledge–Attitude–Practice and Self-Efficacy Questionnaire (AOMH-KAPQ)
Source: Healthcare (Basel). 2026 Aug 19;14(16):2615. doi: 10.3390/healthcare14162615 (PMC13512447; doi:10.3390/healthcare14162615)
Supplement: Supplementary file 1 [file healthcare-14-02615-s001.zip › healthcare-4421858-supplementary.pdf]

Development and Initial Psychometric Evaluation of the Adolescent Oral and Maxillofacial Health Knowledge–Attitude–Practice and Self-Efficacy Questionnaire (AOMH-KAPQ)

Like Zhang, Xue Liu, Lei Cui, Xinyao Yang, Xinyue Hou and Lili Hou

Supplementary Materials

Supplementary Table S1. Item-Level Delphi Results, Delphi-Stage Decisions, and Final Status for All 46 Initial Items

Item	Dimension	Clinical topic	Mean importance	CV	I-CVI	Delphi-stage decision	Final psychometric status	Administered item wording or focus (English translation)
K1	Knowledge	Dentofacial deformity	4.56	0.135	0.944	Retained for formal testing; met all three criteria.	Retained in the final 38-item AOMH-KAPQ.	Long-term mouth breathing, tongue thrusting, or finger sucking does not affect tooth alignment or facial development.
K2	Knowledge	Dentofacial deformity	4.50	0.114	0.944	Retained for formal testing; met all three criteria.	Retained in the final 38-item AOMH-KAPQ.	The best time to correct an anterior crossbite ("underbite") is after all permanent teeth have erupted.
K3	Knowledge	Dentofacial deformity	4.33	0.177	1.000	Retained for formal testing; met all three criteria.	Retained in the final 38-item AOMH-KAPQ.	The only purpose of orthodontic treatment (braces) is aesthetics; it does not help chewing or periodontal health.
K4	Knowledge	Dental trauma	4.39	0.138	1.000	Retained for formal testing; met all three criteria.	Retained in the final 38-item AOMH-KAPQ.	When a tooth is completely knocked out, you should pick it up at once and scrub the root clean with a tissue or soapy water.
K5	Knowledge	Dental trauma	4.22	0.223	1.000	Retained for formal testing; met all three criteria.	Retained in the final 38-item AOMH-KAPQ.	After a tooth is hit, there is no need to see a dentist as long as it is only slightly loose, painless, and not knocked out.
K6	Knowledge	Dental trauma	4.39	0.114	0.889	Retained for formal testing; met all three criteria.	Retained in the final 38-item AOMH-KAPQ.	After a permanent tooth is completely knocked out, the sooner it is replanted/treated the better—ideally at a clinic as soon as possible (e.g., within 30 minutes).
K7	Knowledge	TMD	4.44	0.193	0.889	Retained for formal testing; met all three criteria.	Retained in the final 38-item AOMH-KAPQ.	Jaw-joint clicking when opening the mouth needs no attention at all as long as it is painless.
K8	Knowledge	TMD	4.00	0.192	1.000	Retained for formal testing; met all three criteria.	Retained in the final 38-item AOMH-KAPQ.	If the jaw-joint area is painful or the mouth is hard to open, you should forcefully open wide to "stretch it out."
K9	Knowledge	OSA	4.39	0.159	1.000	Retained for formal testing; met all three criteria.	Retained in the final 38-item AOMH-KAPQ.	Snoring during sleep means good sleep quality (sleeping soundly).
K10	Knowledge	OSA	4.33	0.177	1.000	Retained for formal testing; met all three criteria.	Retained in the final 38-item AOMH-KAPQ.	Only obese adolescents develop sleep-breathing problems such as snoring with pauses/gasping.
K11	Knowledge	Oral ulcer	4.56	0.135	1.000	Retained for formal testing; met all three criteria.	Retained in the final 38-item AOMH-KAPQ.	Mouth ulcers are all caused by "internal heat"; you only need to drink more water and need not see a doctor.
K12	Knowledge	Oral ulcer	4.50	0.114	1.000	Retained for formal testing; met all three criteria.	Retained in the final 38-item AOMH-KAPQ.	If a mouth ulcer at the same site does not heal within 2 weeks (14 days), you should be highly alert and seek care promptly.
K13	Knowledge	Oral ulcer	3.72	0.257	0.722	Provisionally carried forward for formal testing after content and wording review; failed criterion/criteria: mean importance ≥ 4.0, CV < .25, I-CVI ≥ .78.	Retained in the final 38-item AOMH-KAPQ; 2PL a = 1.08 and b = .11.	Common (non-viral) mouth ulcers can be passed to classmates by sharing cups or eating together.
K14	Knowledge	Missing teeth / implant	4.06	0.158	0.722	Provisionally carried forward for formal testing after content and wording review; failed criterion/criteria: I-CVI ≥ .78.	Retained in the final 38-item AOMH-KAPQ; 2PL a = 1.25 and b = .27.	An adolescent (e.g., 14 years old) who loses a permanent tooth to trauma should have a dental implant placed right away.
K15	Knowledge	Missing teeth / implant	4.33	0.158	0.889	Retained for formal testing; met all three criteria.	Retained in the final 38-item AOMH-KAPQ.	Once a dental implant is placed, it does not need careful daily cleaning the way a natural tooth does.
A1	Attitude	General	4.50	0.137	1.000	Retained for formal testing; met all three criteria.	Retained in the final 38-item AOMH-KAPQ.	I think it is very necessary for adolescents to know about oral and maxillofacial conditions (e.g., temporomandibular disorders, dental trauma).

Item	Dimension	Clinical topic	Mean importance	CV	I-CVI	Delphi-stage decision	Final psychometric status	Administered item wording or focus (English translation)
A2	Attitude	Dentofacial deformity / malocclusion	4.50	0.114	1.000	Retained for formal testing; met all three criteria.	Deleted during CFA refinement because of high content overlap with A3 (MI = 12.061).	I think malocclusions such as anterior crossbite ("underbite") not only affect appearance, but may also affect chewing and facial development, and should be examined and evaluated early.
A3	Attitude	Dentofacial deformity	4.83	0.079	1.000	Retained for formal testing; met all three criteria.	Retained in the final 38-item AOMH-KAPQ.	Whether an anterior crossbite ("underbite") needs early intervention should follow a professional's advice, and intervening at the right time is worthwhile.
A4	Attitude	Dental trauma	4.50	0.137	1.000	Retained for formal testing; met all three criteria.	Retained in the final 38-item AOMH-KAPQ.	I believe that if a knocked-out tooth is handled properly (correctly stored and treated promptly), it has a chance of being saved.
A5	Attitude	TMD	3.00	0.302	0.611	Provisionally carried forward for formal testing after content and wording review; failed criterion/criteria: mean importance ≥ 4.0, CV < .25, I-CVI ≥ .78.	Deleted after item analysis because of poor consistency (CITC = .158; alpha after deletion = .901).	I think that jaw-joint clicking ("clicking sounds") usually does not need treatment as long as it is not painful.
A6	Attitude	Dental trauma	4.22	0.223	1.000	Retained for formal testing; met all three criteria.	Retained in the final 38-item AOMH-KAPQ.	Wearing a sports mouthguard during contact sports such as basketball and soccer can effectively reduce the risk of dental injury.
A7	Attitude	TMD	4.33	0.177	1.000	Retained for formal testing; met all three criteria.	Retained in the final 38-item AOMH-KAPQ.	Recurrent jaw-joint clicking, or clicking with pain or limited mouth opening, should be taken seriously and evaluated by a doctor.
A8	Attitude	TMD	4.44	0.159	1.000	Retained for formal testing; met all three criteria.	Retained in the final 38-item AOMH-KAPQ.	I believe stress (e.g., exam pressure) can worsen jaw clenching/grinding and thereby cause jaw soreness or pain.
A9	Attitude	OSA	4.50	0.114	1.000	Retained for formal testing; met all three criteria.	Retained in the final 38-item AOMH-KAPQ.	Marked snoring, or daytime sleepiness and poor concentration, may signal a health problem and should be checked by a doctor.
A10	Attitude	Oral ulcer	4.39	0.138	1.000	Retained for formal testing; met all three criteria.	Retained in the final 38-item AOMH-KAPQ.	Spicy, very hot, or hard foods should be avoided during a mouth ulcer, as this helps relieve pain and promote healing.
A11	Attitude	Oral ulcers	3.61	0.287	0.778	Provisionally carried forward for formal testing after content and wording review; failed criterion/criteria: mean importance ≥ 4.0, CV < .25, I-CVI ≥ .78.	Deleted after item analysis because of poor consistency (CITC = .156; alpha after deletion = .902).	I think mouth ulcers will recover by drinking more water and do not need medical care.
A12	Attitude	Missing teeth / implant	4.11	0.219	1.000	Retained for formal testing; met all three criteria.	Retained in the final 38-item AOMH-KAPQ.	If an adolescent has a missing permanent tooth, they should seek early evaluation for restoration even if eating and daily life are not yet affected.
P1	Practice	General / cross-cutting prevention	4.39	0.114	1.000	Retained for formal testing; met all three criteria.	Deleted after EFA/CFA review because of low factor loading (.346/.239; standardized loading < .40).	On average, I brush my teeth at least twice a day (morning and evening).
P2	Practice	General prevention	4.50	0.157	0.889	Retained for formal testing; met all three criteria.	Retained in the final 38-item AOMH-KAPQ.	I use dental floss or an interdental brush to clean between my teeth.
P3	Practice	General prevention	4.17	0.236	0.944	Retained for formal testing; met all three criteria.	Retained in the final 38-item AOMH-KAPQ.	I am careful to avoid chewing food on only one side for a long time.
P4	Practice	General / oral habit management	3.56	0.277	0.611	Provisionally carried forward for formal testing after content and wording review; failed criterion/criteria: mean importance ≥ 4.0, CV < .25, I-CVI ≥ .78.	Deleted after item analysis because the reverse-scored item showed inconsistent direction (CITC = -.059).	I have habits such as biting pens, biting my lips, picking the skin of my lips, or long-term mouth breathing.
P5	Practice	General prevention	4.06	0.179	0.778	Provisionally carried forward for formal testing after content and wording review; failed criterion/criteria: I-CVI ≥ .78.	Retained in the final 38-item AOMH-KAPQ; loading = .621 and CITC = .400.	During contact sports (e.g., basketball, soccer), I wear a sports mouthguard.
P6	Practice	Dental trauma	4.28	0.176	0.944	Retained for formal testing; met all three criteria.	Retained in the final 38-item AOMH-KAPQ.	After a tooth is hit (even if only loosened or slightly painful), I tell my parents right away and see a dentist as soon as possible.

Item	Dimension	Clinical topic	Mean importance	CV	I-CVI	Delphi-stage decision	Final psychometric status	Administered item wording or focus (English translation)
P7	Practice	TMD / stress-related oral behavior	3.50	0.177	0.722	Provisionally carried forward for formal testing after content and wording review; failed criterion/criteria: mean importance ≥ 4.0, I-CVI ≥ .78.	Deleted after item analysis because the reverse-scored item showed inconsistent direction (CITC = -.203).	When I feel stressed (e.g., before examinations), I unconsciously clench or grind my teeth.
P8	Practice	TMD	4.56	0.112	1.000	Retained for formal testing; met all three criteria.	Retained in the final 38-item AOMH-KAPQ.	If my jaw joint clicks with pain or difficulty opening the mouth for more than 2 weeks, I tell my parents and ask to see a doctor.
P9	Practice	TMD self-care	3.22	0.293	0.778	Provisionally carried forward for formal testing after content and wording review; failed criterion/criteria: mean importance ≥ 4.0, CV < .25, I-CVI ≥ .78.	Deleted after EFA because of salient cross-loading (.382/.400).	When I feel soreness in the jaw-joint area (in front of the ear), I try using a warm towel compress to relax it.
P10	Practice	OSA	3.94	0.203	0.611	Provisionally carried forward for formal testing after content and wording review; failed criterion/criteria: mean importance ≥ 4.0, I-CVI ≥ .78.	Retained in the final 38-item AOMH-KAPQ; loading = .642 and CITC = .733.	When I notice I snore heavily, or classmates tell me so and I also feel sleepy during the day, I tell my parents and ask to see a doctor.
P11	Practice	Oral ulcer	4.67	0.104	1.000	Retained for formal testing; met all three criteria.	Retained in the final 38-item AOMH-KAPQ.	If I find a mouth ulcer at the same site lasting more than 2 weeks, I tell my parents and ask to see a doctor.
P12	Practice	Oral ulcers / oral hygiene during symptoms	3.67	0.281	0.722	Provisionally carried forward for formal testing after content and wording review; failed criterion/criteria: mean importance ≥ 4.0, CV < .25, I-CVI ≥ .78.	Deleted after item analysis because the reverse-scored item showed inconsistent direction (CITC = -.190).	When I have a mouth ulcer, I reduce how often I brush my teeth because of pain, or I brush only briefly.
P13	Practice	Oral ulcer	4.56	0.112	0.889	Retained for formal testing; met all three criteria.	Retained in the final 38-item AOMH-KAPQ.	When I have a mouth ulcer, I deliberately avoid spicy, very hot, or hard foods.
P14	Practice	Missing teeth / implant	4.61	0.132	1.000	Retained for formal testing; met all three criteria.	Retained in the final 38-item AOMH-KAPQ.	If a doctor recommends a dental implant, I find out about the post-implant care (daily cleaning and regular follow-up) and am willing to follow it.
SE1	Self-efficacy	General	4.56	0.135	1.000	Retained for formal testing; met all three criteria.	Retained in the final 38-item AOMH-KAPQ.	Even when busy or tired with schoolwork, I can keep brushing carefully twice a day (at least 2 minutes each time).
SE2	Self-efficacy	General	4.50	0.137	1.000	Retained for formal testing; met all three criteria.	Retained in the final 38-item AOMH-KAPQ.	I can recognize and correct (or avoid) harmful habits such as lip-biting and one-sided chewing.
SE3	Self-efficacy	Dental trauma	4.56	0.155	1.000	Retained for formal testing; met all three criteria.	Retained in the final 38-item AOMH-KAPQ.	If a sudden dental injury occurs (a tooth loosened or knocked out), I am confident I can stay calm, handle it correctly, and seek care immediately.
SE4	Self-efficacy	General care-seeking	4.61	0.132	1.000	Retained for formal testing; met all three criteria.	Retained in the final 38-item AOMH-KAPQ.	When I notice an oral/maxillofacial abnormality (e.g., jaw clicking, a long-unhealed ulcer), I am confident I can overcome delay or fear and ask my parents to take me to a doctor.
SE5	Self-efficacy	Appliance/orthodontic	4.67	0.104	1.000	Retained for formal testing; met all three criteria.	Retained in the final 38-item AOMH-KAPQ.	If a doctor advises me to wear an appliance (e.g., braces, a sports mouthguard), I am confident I can put up with the inconvenience and wear it as prescribed.

Note. Mean importance was calculated from 18 ratings on a 1–5 scale; CV is the sample standard deviation divided by the mean; I-CVI is the proportion of 18 experts assigning a relevance rating of 3 or 4. Prespecified retention required mean importance ≥ 4.0, CV < .25, and I-CVI ≥ .78, using unrounded values. Thirty-six items met all three criteria. Ten items (K13, K14, A5, A11, P4, P5, P7, P9, P10, and P12) failed at least one criterion and were carried into formal testing provisionally rather than accepted as final. Six of those provisional items were subsequently removed. The retained provisional items had nonredundant content and acceptable later-stage functioning: K13 (2PL a = 1.08, b = .11), K14 (a = 1.25, b = .27), P5 (loading = .621, CITC = .400), and P10 (loading = .642, CITC = .733). The CITCs were calculated against the final nine-item practice-domain score in the full sample (N = 1,006). A2 and P1 met the Delphi criteria but were removed after large-sample testing. Delphi-stage decision and final psychometric status are therefore reported separately.

Supplementary Table S2. Item-Source and Specificity Mapping for the Retained 38 Items

Item	Topic	Development source category	Clinical scenario or cue	Group-level educational need or construct represented
K1	Dentofacial deformity / malocclusion	Literature review; orthodontic clinical Q&A; Delphi and cognitive review	Mouth breathing, tongue thrusting, or finger sucking as cues for alignment/facial-development risk	Correct misconception and prompt early risk recognition
K2	Dentofacial deformity / malocclusion	Literature review; orthodontic clinical Q&A; Delphi and cognitive review	Anterior crossbite and timing of professional assessment	Explain time-sensitive consultation rather than waiting until all permanent teeth erupt
K3	Dentofacial deformity / malocclusion	Literature review; orthodontic clinical Q&A; Delphi and cognitive review	Orthodontic treatment framed beyond appearance	Connect malocclusion care with chewing and periodontal health
K4	Dental trauma	Literature review; dental-trauma clinical Q&A; Delphi and cognitive review	Completely knocked-out tooth and incorrect root scrubbing	Teach emergency handling of an avulsed tooth
K5	Dental trauma	Literature review; dental-trauma clinical Q&A; Delphi and cognitive review	Slight loosening or pain after impact	Encourage parent communication and dental evaluation after injury
K6	Dental trauma	Literature review; dental-trauma clinical Q&A; Delphi and cognitive review	Time-critical replantation of an avulsed permanent tooth	Prioritize urgent care-seeking despite lower item discrimination
K7	TMD	Literature review; TMD clinical Q&A; Delphi and cognitive review	Jaw-joint clicking without pain	Differentiate harmless symptoms from symptoms requiring monitoring
K8	TMD	Literature review; TMD clinical Q&A; Delphi and cognitive review	Jaw pain or limited opening and harmful forced stretching	Correct unsafe self-management
K9	OSA-related manifestations	Literature review; sleep-breathing clinical Q&A; Delphi and cognitive review	Snoring interpreted as good sleep quality	Improve recognition of abnormal sleep-breathing signals
K10	OSA-related manifestations	Literature review; sleep-breathing clinical Q&A; Delphi and cognitive review	Misconception that only obese adolescents develop sleep-breathing problems	Broaden risk recognition beyond weight status alone
K11	Oral ulcers	Literature review; oral-mucosal clinical Q&A; Delphi and cognitive review	Mouth ulcers attributed only to internal heat	Discourage delayed care based on folk explanations alone
K12	Oral ulcers	Literature review; oral-mucosal clinical Q&A; Delphi and cognitive review	Ulcer persisting at the same site for more than 2 weeks	Trigger timely care-seeking for persistent lesions
K13	Oral ulcers	Literature review; oral-mucosal clinical Q&A; Delphi and cognitive review	Misconception that common non-viral ulcers are contagious	Reduce unnecessary avoidance and clarify common ulcer knowledge
K14	Missing permanent teeth / restorative consultation	Literature review; restorative/implant clinical Q&A; Delphi and cognitive review	Adolescent permanent-tooth loss and misconception of immediate implant placement	Clarify that implant timing requires professional assessment and growth consideration
K15	Missing permanent teeth / restorative consultation	Literature review; restorative/implant clinical Q&A; Delphi and cognitive review	Maintenance misconception after implant placement	Teach cleaning and follow-up needs for restorative treatment
A1	General / cross-cutting prevention	Literature review; clinical Q&A across subspecialties; Delphi and cognitive review	Perceived need to know about oral and maxillofacial conditions	Assess overall value placed on oral and maxillofacial health knowledge
A3	Dentofacial deformity / malocclusion	Literature review; orthodontic clinical Q&A; Delphi and cognitive review	Professional advice and timing for anterior crossbite intervention	Strengthen acceptance of time-appropriate orthodontic consultation
A4	Dental trauma	Literature review; dental-trauma clinical Q&A; Delphi and cognitive review	Possibility of saving a knocked-out tooth if handled correctly	Build positive expectancy for emergency action
A6	Dental trauma	Literature review; sports-injury clinical Q&A; Delphi and cognitive review	Mouthguard use during contact sport	Strengthen risk perception and preventive attitude
A7	TMD	Literature review; TMD clinical Q&A; Delphi and cognitive review	Recurrent clicking, pain, or limited opening	Encourage attention to symptoms that warrant evaluation

Item	Topic	Development source category	Clinical scenario or cue	Group-level educational need or construct represented
A8	TMD	Literature review; TMD clinical Q&A; Delphi and cognitive review	Exam stress, clenching/grinding, and jaw soreness	Link stress-related behaviors with symptom management
A9	OSA-related manifestations	Literature review; sleep-breathing clinical Q&A; Delphi and cognitive review	Marked snoring, daytime sleepiness, and poor concentration	Promote concern and parent communication for possible sleep-breathing problems
A10	Oral ulcers	Literature review; oral-mucosal clinical Q&A; Delphi and cognitive review	Food choices during mouth ulcer episodes	Support symptom-relief behavior during ulcer healing
A12	Missing permanent teeth / restorative consultation	Literature review; restorative clinical Q&A; Delphi and cognitive review	Missing permanent tooth even when daily life is not affected	Promote early evaluation for restorative options
P2	General / cross-cutting prevention	Literature review; preventive oral-health Q&A; Delphi and cognitive review	Interdental cleaning with floss or an interdental brush	Identify daily preventive behavior needing instruction
P3	TMD / oral habit management	Literature review; TMD and oral-habit Q&A; Delphi and cognitive review	Long-term unilateral chewing	Assess habit-management behavior
P5	Dental trauma	Literature review; sports-injury clinical Q&A; Delphi and cognitive review	Mouthguard use during contact sport	Identify adoption of sports-trauma prevention
P6	Dental trauma	Literature review; dental-trauma clinical Q&A; Delphi and cognitive review	Tooth impact with looseness or pain	Assess parent communication and prompt care-seeking after injury
P8	TMD	Literature review; TMD clinical Q&A; Delphi and cognitive review	Jaw clicking with pain or opening difficulty lasting more than 2 weeks	Assess symptom monitoring and care-seeking
P10	OSA-related manifestations	Literature review; sleep-breathing clinical Q&A; Delphi and cognitive review	Heavy snoring with daytime sleepiness	Assess parent communication and care-seeking intention/behavior
P11	Oral ulcers	Literature review; oral-mucosal clinical Q&A; Delphi and cognitive review	Same-site ulcer lasting more than 2 weeks	Assess communication and care-seeking for persistent lesions
P13	Oral ulcers	Literature review; oral-mucosal clinical Q&A; Delphi and cognitive review	Spicy, very hot, or hard foods during an ulcer	Assess symptom-relief self-care behavior
P14	Missing permanent teeth / restorative consultation	Literature review; restorative/implant clinical Q&A; Delphi and cognitive review	Doctor-recommended implant/restorative care and maintenance	Assess context-dependent willingness to learn and follow maintenance advice
SE1	General / cross-cutting self-management	Literature review; preventive oral-health Q&A; Delphi and cognitive review	Brushing carefully when busy or tired	Assess confidence in maintaining routine behavior under school pressure
SE2	General / oral habit management	Literature review; oral-habit Q&A; Delphi and cognitive review	Recognizing and correcting lip-biting or one-sided chewing	Assess confidence in habit recognition and correction
SE3	Dental trauma	Literature review; dental-trauma clinical Q&A; Delphi and cognitive review	Sudden dental injury	Assess confidence to stay calm, act correctly, and seek care
SE4	General / abnormality recognition	Literature review; clinical Q&A across subspecialties; Delphi and cognitive review	Jaw clicking or long-unhealed ulcer noticed by the adolescent	Assess confidence to overcome delay/fear and ask parents for medical care
SE5	Missing permanent teeth / appliance adherence	Literature review; orthodontic/restorative clinical Q&A; Delphi and cognitive review	Wearing an appliance, braces, or mouthguard as prescribed	Assess confidence in adherence despite inconvenience

Note. This table maps each retained item to the development source categories and clinical scenario used during item generation; it is not intended as an item-by-item citation list.

Supplementary Table S3. Two-Parameter (2PL) IRT Item Parameters for the 15 Knowledge Items (Full Sample, *N* = 1,006)

Item	Topic	Discrimination *a* (SE)	Difficulty *b* (SE)
K1	Dentofacial deformity	1.40 (0.13)	−0.79 (0.08)
K2	Dentofacial deformity	1.47 (0.14)	0.53 (0.07)

Item	Topic	Discrimination *a* (SE)	Difficulty *b* (SE)
K3	Dentofacial deformity	2.25 (0.20)	−0.83 (0.06)
K4	Dental trauma	1.07 (0.11)	0.66 (0.09)
K5	Dental trauma	3.25 (0.32)	−0.91 (0.06)
K6	Dental trauma	0.58 (0.08)	−1.25 (0.20)
K7	TMD	3.58 (0.37)	−0.83 (0.06)
K8	TMD	2.55 (0.23)	−0.63 (0.06)
K9	OSA manifestations	2.57 (0.23)	−0.78 (0.06)
K10	OSA manifestations	2.47 (0.22)	−0.65 (0.06)
K11	Oral ulcers	2.69 (0.24)	−0.87 (0.06)
K12	Oral ulcers	0.55 (0.09)	−1.82 (0.29)
K13	Oral ulcers	1.08 (0.11)	0.11 (0.07)
K14	Missing teeth / restorative	1.25 (0.12)	0.27 (0.07)
K15	Missing teeth / restorative	2.44 (0.22)	−1.08 (0.07)

Note. Two-parameter logistic (2PL) model fitted to the 15 dichotomously scored knowledge items in the full sample (N = 1,006), estimated with the *mirt* package. a = item discrimination; b = item difficulty; standard errors in parentheses. Model fit (M2): RMSEA = .079, CFI = .950, TLI = .942; empirical reliability = .817. K6 and K12 (keyed True) had the lowest discrimination (a = 0.58 and 0.55) but were retained for their safety-relevant content (dental-trauma replantation timing and persistent-ulcer care-seeking). Most difficulty (b) values were below 0, indicating that the knowledge items were generally easy. Because the knowledge dimension spans several topics, these results are interpreted as exploratory item-level information rather than as evidence of strict unidimensionality.

Supplementary. Scoring instructions for the AOMH-KAPQ

Knowledge (K; 15 items). Each item is answered True / False / Not sure and scored dichotomously as 1 (correct) or 0 (incorrect); a "Not sure" response is scored 0. Two items are keyed *True* (K6 and K12, which state correct safety facts); the remaining 13 items are misconception statements keyed *False*. The knowledge score is the sum of the 15 items (range 0–15), with higher scores indicating more accurate knowledge.

Attitude (A; 9 items), Practice (P; 9 items), and Self-efficacy (SE; 5 items). Each item is rated on a 5-point Likert scale scored 1–5 in the direction of its response anchors (attitude, 1 = strongly disagree to 5 = strongly agree; practice, 1 = never to 5 = always; self-efficacy, 1 = no confidence at all to 5 = very confident). The final version contains no reverse-scored items—all reverse-worded items were removed during psychometric testing—so no items require recoding. Each dimension score is the sum of its items (attitude 9–45, practice 9–45, self-efficacy 5–25), or the item mean (1–5) if a common 1–5 metric is preferred; higher scores indicate a more favorable attitude, a higher practice-domain score, and higher self-efficacy. Because respondents who had not encountered a conditional scenario were instructed to answer how they would respond, the practice score combines reported behavior with intended conditional responses and should not be interpreted as observed behavior frequency alone.

Practice item groupings. In this sample, B1 grouped condition-responsive self-care and care-seeking items (P6, P8, P10, P11, P13, and P14), whereas B2 grouped routine preventive practices (P2, P3, and P5). These labels describe observed item groupings; separate subscale scoring is not recommended at this stage because B1 and B2 were strongly related and B2 showed limited convergent/discriminant evidence.

Use of scores. Because knowledge is an objective 0/1 test whereas attitude, practice, and self-efficacy are self-reported Likert ratings, the four domain scores should be reported separately and a single overall total score is not recommended. No low/moderate/high categories, diagnostic cutoff, or individual clinical-risk threshold has been developed or validated. At the current stage, scores are most appropriate for research and group-level educational needs assessment in populations similar to the study sample. Responsiveness has not been established, so pre–post score changes should be treated as exploratory.

Supplementary Table S4. The AOMH-KAPQ (final 38 items)

English translation of the administered Chinese items.

Knowledge (15 items): respondents judge each statement True / False / Not sure (1 = correct, 0 = incorrect). K6 and K12 are keyed True; the others are misconception statements keyed False.

Code	Item
K1	Long-term mouth breathing, tongue thrusting, or finger sucking does not affect tooth alignment or facial development.
K2	The best time to correct an anterior crossbite ("underbite") is after all permanent teeth have erupted.
K3	The only purpose of orthodontic treatment (braces) is aesthetics; it does not help chewing or periodontal health.
K4	When a tooth is completely knocked out, you should pick it up at once and scrub the root clean with a tissue or soapy water.
K5	After a tooth is hit, there is no need to see a dentist as long as it is only slightly loose, painless, and not knocked out.
K6	After a permanent tooth is completely knocked out, the sooner it is replanted/treated the better—ideally at a clinic as soon as possible (e.g., within 30 minutes).
K7	Jaw-joint clicking when opening the mouth needs no attention at all as long as it is painless.
K8	If the jaw-joint area is painful or the mouth is hard to open, you should forcefully open wide to "stretch it out."
K9	Snoring during sleep means good sleep quality (sleeping soundly).
K10	Only obese adolescents develop sleep-breathing problems such as snoring with pauses/gasping.
K11	Mouth ulcers are all caused by "internal heat"; you only need to drink more water and need not see a doctor.
K12	If a mouth ulcer at the same site does not heal within 2 weeks (14 days), you should be highly alert and seek care promptly.
K13	Common (non-viral) mouth ulcers can be passed to classmates by sharing cups or eating together.
K14	An adolescent (e.g., 14 years old) who loses a permanent tooth to trauma should have a dental implant placed right away.
K15	Once a dental implant is placed, it does not need careful daily cleaning the way a natural tooth does.

Attitude (9 items): 1 = strongly disagree … 5 = strongly agree.

Code	Item
A1	I think it is very necessary for adolescents to know about oral and maxillofacial conditions (e.g., temporomandibular disorders, dental trauma).
A3	Whether an anterior crossbite ("underbite") needs early intervention should follow a professional's advice, and intervening at the right time is worthwhile.
A4	I believe that if a knocked-out tooth is handled properly (correctly stored and treated promptly), it has a chance of being saved.
A6	Wearing a sports mouthguard during contact sports such as basketball and soccer can effectively reduce the risk of dental injury.
A7	Recurrent jaw-joint clicking, or clicking with pain or limited mouth opening, should be taken seriously and evaluated by a doctor.
A8	I believe stress (e.g., exam pressure) can worsen jaw clenching/grinding and thereby cause jaw soreness or pain.

Code	Item
A9	Marked snoring, or daytime sleepiness and poor concentration, may signal a health problem and should be checked by a doctor.
A10	Spicy, very hot, or hard foods should be avoided during a mouth ulcer, as this helps relieve pain and promote healing.
A12	If an adolescent has a missing permanent tooth, they should seek early evaluation for restoration even if eating and daily life are not yet affected.

Practice (9 items): 1 = never … 5 = always (past 6 months; if not encountered, answer as you would).

Code	Item
P2	I use dental floss or an interdental brush to clean between my teeth.
P3	I am careful to avoid chewing food on only one side for a long time.
P5	During contact sports (e.g., basketball, soccer), I wear a sports mouthguard.
P6	After a tooth is hit (even if only loosened or slightly painful), I tell my parents right away and see a dentist as soon as possible.
P8	If my jaw joint clicks with pain or difficulty opening the mouth for more than 2 weeks, I tell my parents and ask to see a doctor.
P10	When I notice I snore heavily, or classmates tell me so and I also feel sleepy during the day, I tell my parents and ask to see a doctor.
P11	If I find a mouth ulcer at the same site lasting more than 2 weeks, I tell my parents and ask to see a doctor.
P13	When I have a mouth ulcer, I deliberately avoid spicy, very hot, or hard foods.
P14	If a doctor recommends a dental implant, I find out about the post-implant care (daily cleaning and regular follow-up) and am willing to follow it.

Self-efficacy (5 items): 1 = no confidence at all … 5 = very confident.

Code	Item
SE1	Even when busy or tired with schoolwork, I can keep brushing carefully twice a day (at least 2 minutes each time).
SE2	I can recognize and correct (or avoid) harmful habits such as lip-biting and one-sided chewing.
SE3	If a sudden dental injury occurs (a tooth loosened or knocked out), I am confident I can stay calm, handle it correctly, and seek care immediately.
SE4	When I notice an oral/maxillofacial abnormality (e.g., jaw clicking, a long-unhealed ulcer), I am confident I can overcome delay or fear and ask my parents to take me to a doctor.
SE5	If a doctor advises me to wear an appliance (e.g., braces, a sports mouthguard), I am confident I can put up with the inconvenience and wear it as prescribed.

Supplementary Table S5. CFA Estimator, Parameterization, and Alternative-Model Sensitivity Analyses (CFA Half, N = 503)

Model	Estimator / specification	Robust CFI	Robust TLI	Robust RMSEA (95% CI)	SRMR	Convergence / interpretation
Attitude, one factor	WLSMV, delta	.946	.928	.137 [.125, .150]	.030	Converged; admissible.
Attitude, one factor	WLSMV, theta	.946	.928	.137 [.125, .150]	.030	Converged; admissible.
Attitude, one factor	ULSMV, delta	.944	.926	.139 [.128, .151]	.026	Converged; admissible.
Attitude, one factor	MLR, continuous	.952	.936	.110 [.089, .131]	.034	Converged; admissible. Secondary continuous-item sensitivity analysis.
Practice, two factors + P6~~P8	WLSMV, delta	.913	.875	.145 [.129, .161]	.051	Converged; retained as a same-sample refinement. Fit improved over the two-factor model without the residual covariance (scaled Δχ² = 60.28, Δdf = 1, p < .001); independent replication is required.
Practice, two factors + P6~~P8	WLSMV, theta	.913	.875	.145 [.129, .161]	.051	Converged; admissible.
Practice, two factors + P6~~P8	ULSMV, delta	.912	.874	.145 [.130, .161]	.049	Converged; admissible.
Practice, two factors + P6~~P8	MLR, continuous	.945	.921	.096 [.077, .115]	.041	Converged; admissible. Secondary continuous-item sensitivity analysis.
Practice, one factor	WLSMV, delta	.873	.831	.168 [.153, .183]	.064	Converged; fit was poorer than the two-factor model without a residual covariance.
Practice, two factors without residual covariance	WLSMV, delta	.897	.857	.154 [.139, .170]	.053	Converged; fit improved over the one-factor model (scaled Δχ² = 50.79, Δdf = 1, p < .001).
Practice bifactor	WLSMV, delta	—	—	—	—	Did not converge to an admissible solution; fit indices were not interpreted.
Joint four-factor	WLSMV, delta	.905	.893	.094 [.088, .100]	.047	Converged; admissible.
Joint four-factor	WLSMV, theta	.905	.893	.094 [.088, .100]	.047	Converged; admissible.
Joint four-factor	ULSMV, delta	.901	.888	.096 [.090, .102]	.045	Converged; admissible.
Joint four-factor	MLR, continuous	.945	.937	.060 [.053, .067]	.044	Converged; admissible. Secondary continuous-item sensitivity analysis.
Attitude + A7~~A8	WLSMV, delta	.945	.924	.141 [.129, .154]	.030	Converged; admissible. No improvement in robust ordinal RMSEA.
Attitude + A4~~A6	WLSMV, delta	.946	.925	.140 [.127, .152]	.030	Converged; admissible. No improvement in robust ordinal RMSEA.
Attitude + both residuals	WLSMV, delta	.945	.921	.144 [.131, .157]	.030	Converged; admissible. No improvement in robust ordinal RMSEA.
Attitude content bifactor	WLSMV, delta	—	—	—	—	Did not converge; fit indices were not interpreted.

Note. WLSMV and ULSMV treated the five response categories as ordered; MLR treated them as continuous. CFI, TLI, and RMSEA are robust values; SRMR is the standard value. Delta and theta are WLSMV parameterizations. The practice comparisons were conducted sequentially: the EFA-derived two-factor model without a residual covariance was compared with the one-factor model, after which the P6–P8 residual covariance was added. P6 and P8 both involve informing a parent and seeking professional care. Because that residual covariance was evaluated in the same CFA half, it is a same-sample refinement and requires independent replication. The nine-item attitude model was likewise obtained after A2 was removed using a modification index in the same CFA half; its fit also requires independent confirmation. The attitude residual alternatives represented local dependence between the two TMD-related items (A7–A8) and the two dental-trauma-related items (A4–A6). The orthogonal bifactor model specified a general attitude factor, a professional-evaluation/care group factor (A3, A7, A9, A12), and a prevention/management-belief group factor (A4, A6, A8, A10), with A1 loading only on the general factor. These were same-sample exploratory sensitivity analyses and do not replace independent cross-validation. CFI = comparative fit index; MLR = robust maximum likelihood; RMSEA = root mean square error of approximation; SRMR = standardized root mean square residual; TLI = Tucker–Lewis index; ULSMV = unweighted least squares mean- and variance-adjusted; WLSMV = weighted least squares mean- and variance-adjusted.

Supplementary Table S6. Convergent and Discriminant Validity in the Joint Measurement Model (CFA Half, N = 503)

Dimension	AVE	CR	A	B1	B2	SE
Attitude (A)	.732	.961	.856			
Practice—condition-responsive self-care and care-seeking (B1)	.679	.927	.600	.824		
Practice—routine preventive practices (B2)	.457	.712	.427	.783	.676	
Self-efficacy (SE)	.709	.924	.412	.537	.586	.842

Note. Diagonal entries are √AVE; values below the diagonal are inter-factor correlations. HTMT for B1–B2 was .800. B2 AVE was .457 and √AVE for B2 (.676) was below the B1–B2 correlation (.783), so B1 and B2 should be treated as provisional descriptive components rather than independently validated subscales. AVE = average variance extracted; CR = composite reliability; HTMT = heterotrait–monotrait ratio.

Supplementary Table S7. Measurement Invariance Across Self-Reported Gender and Age Groups (CFA Half, N = 503)

Grouping variable	Invariance level	Comparison	ΔCFI	ΔRMSEA	Decision
Self-reported gender	Metric/weak invariance	Metric vs. configural	.000	.002	Pass
Self-reported gender	Scalar/strong invariance	Scalar vs. metric	.005	.003	Pass
Age group	Metric/weak invariance	Metric vs. configural	.002	.001	Pass
Age group	Scalar/strong invariance	Scalar vs. metric	.007	.002	Pass

Note. Pass criteria were ΔCFI ⩽ .010 and ΔRMSEA ⩽ .015. The analysis concerned the tested Likert-domain joint model (A, B1, B2, and SE); the knowledge domain was not included. These relative invariance results are conditional on the model's provisional structure and mixed absolute fit. They do not establish equivalence in other geographic or cultural populations.

Supplementary Table S8. Interpretive Summary of Knowledge-Domain 2PL Results (Full Sample, N = 1,006; Per-Item Estimates and Standard Errors in Table S3)

IRT component	Result	Interpretation
2PL model fit for the knowledge domain	RMSEA = .079; CFI = .950; TLI = .942	Approximate fit for exploratory item-level evidence; not proof of strict unidimensionality
Empirical reliability	.817	Adequate reliability for the dichotomously scored knowledge domain
Item discrimination range	a = 0.550–3.580; most items > 1	Most items showed moderate-to-high discrimination
Lowest discrimination	K12: a = 0.550; K6: a = 0.577	Both safety-relevant items require monitoring in independent samples
Item difficulty range	b = −1.816 to 0.660; most items < 0	The relatively easy set provides less information at the high-knowledge end
